# Complications of harvesting a connective tissue graft from the palate. A retrospective study and description of a new technique

**DOI:** 10.4317/jced.54337

**Published:** 2017-12-01

**Authors:** Luis-Antonio Aguirre-Zorzano, Ana M. García-De La Fuente, Ruth Estefanía-Fresco, Xabier Marichalar-Mendía

**Affiliations:** 1 Professor in Periodontics. Department of Stomatology II. Director of the Master’s Degree on Periodontics and Osteointegration. (UPV/EHU); 2 Assistant Professor. Department of Stomatology II. Master’s Degree on Periodontics and Osteointegration. (UPV/EHU); 3 PhD in Dentistry. Associate Teacher. Department of Stomatology II. Master’s Degree on Periodontics and Osteointegration. (UPV/EHU); 4 Ph in Biology. Master´s Degree on Oral Pathology (UPV/EHU). Department of Stomatology II. University of the Basque Country (UPV/EHU)

## Abstract

**Background:**

Connective tissue graft (CTG) is considered as the gold standard for the treatment of gingival recessions (GR). There are few studies assessing the complications that can arise in the donor site when harvesting a connective tissue graft (CTG) and how the harvesting technique can influence those complications.

**Material and Methods:**

A retrospective clinical study was carried out in order to compare the complications observed in 40 patients with Miller class I, II and III GR ≥ 3 mm, after using the trap-door technique (TD) in the control group and a newly described technique, the “UPV/EHU technique”, in the test group. Patients were consecutively allocated to each treatment group. Patients were monitored 14 days after surgery in order to evaluate post-operative complications in the donor site: presence of pain (P), bleeding (B), infection (I) and necrosis > 30%.

**Results:**

Although morbidity was observed in both groups, it was less important in the test group (no pain and minimal pain in 30% and 35% of the cases, respectively, and absence of bleeding or infection and necrosis >30% in only 5% of the cases).

**Conclusions:**

Within the limits of this study, this newly described “UPV/EHU technique” should be considered as a treatment option when harvesting a CTG, with minimal morbidity for patients.

** Key words:**Connective tissue graft, pain, gingival recessions, wound healing, cosmetic periodontal plastic surgery, trap-door technique, “UPV/EHU technique”.

## Introduction

Gingival recessions (GR) are a common finding in the population, and their treatment is part of the daily practice in the periodontal clinic. Several treatment techniques have been proposed, which can be divided into pedicle and free grafts. The latter can also be classified as free gingival grafts (FGG) or as connective tissue grafts (CTG). Other treatment options include soft tissue substitutes or regenerative therapies.

In 1968, Sullivan & Atkins (1) described the FGG technique, which was an easy-to-perform technique that proved effective to achieve keratinized tissue, but was not so predictable in terms of root coverage, obtained poor aesthetic results and was associated with a high postoperative morbidity, due to the graft harvesting technique, which caused a wound in the donor site that would heal by secondary intention.

Aiming to minimize that morbidity, several authors proposed harvesting only the connective tissue (CT), by means of different techniques: the trap-door technique (TD)(2), the parallel incisions technique (PI)(3), and the single incision technique (SI) (4), which was later on modified by Lorenzana and Allen (5) and by Kumar *et al.* (6). According to scientific evidence (7), the CTG is the gold standard in the treatment of GR. However, complications can arise after harvesting the graft, and the harvesting technique could be of importance. Although studies on that subject have been carried out, they are very heterogeneous (8-19).

**Figure 1:**
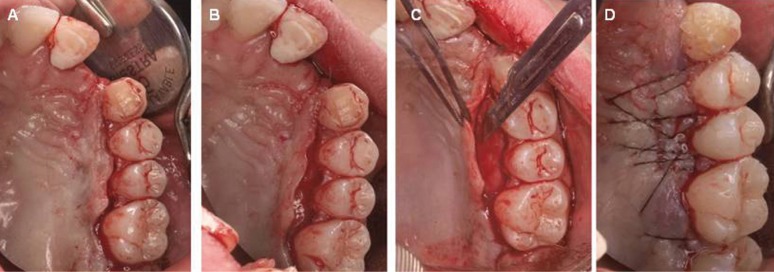
Harvesting the connective tissue graft by means of the UPV/EHU technique. A and B: intrasulcular incision preserving the papillae in the interproximal spaces. C: Full Thickness Flap (FTF) dissected with a 15c scalpel, holding the flap with a tissues forceps to harvest underlying CTG D: Suture and closure of the flap.

The aim of the present study was to assess and compare the complications observed in the donor site after harvesting a graft from the palate performing either the TD technique or our newly described technique, the “UPV/EHU technique” (CTG by partial dissection of a full thickness flap) (Fig. 1).

## Material and Methods

A parallel, retrospective clinical study, was performed to evaluate the healing and the morbidity after harvesting a CTG by means of the TD technique (control group) or a new technique, the “UPV/EHU technique” (test group). No stents were used in any of the groups.

40 patients took part in this study. The patients presented with Miller class I, II and III (20) GR ≥ 3 mm, and were recruited from the Master’s Degree on Periodontology and Osteointegration of the University of the Basque Country (UPV/EHU), between Oc-tober 2013 and November 2014. All of them were properly informed and signed a written informed consent. This study protocol was approved by the Ethics Committee of the UPV/EHU (Ref. CEISH/323/2015).

All patients had to meet the following inclusion criteria in order to take part in this clinical study: healthy patients aged 18 or older, presenting with single or multiple Miller Class I, II or III gingival recessions ≥3 mm, with no systemic contraindications for periodontal surgery, who did not take any medication that could interfere with the healing of periodontal tissues and who had not taken any drugs or medication for the last 6 months; high standards of oral hygiene with a full-mouth plaque score (FMPS) and a full-mouth bleeding score (FMBS) ≤ 20% and self-reported smoking of ≤ 10 cigarettes/day. Patients were excluded from the study when their gingival recessions were associated to dental caries or when they were located in molar teeth.

All patients underwent a presurgical phase consisting on a complete anamnesis and a dental and a periodontal clinical exam. In the periodontal exam, a complete periodontal chart was filled, plaque (21) and bleeding (22) indexes were assessed, and a radiographic exam was carried out (panoramic and periapical radiographs). As all of them were periodontal patients, they underwent a hygienic phase (motivation and oral hygiene instructions, and scaling and root planning (SRP)), and were subsequently enrolled in a supportive periodontal treatment program (SPT). Oral hygiene instructions (Stillman´s technique)(23) were given to the patients in order to eliminate wrong habits of traumatic tooth brushing.

With respect to the surgical phase, patients were consecutively allocated to the control group (TD) (2) or to the test group (“UPV/EHU technique”). All surgical procedures were performed by an experimented clinician (L.A.A.-Z.) in periodontal plastic surgery. Clinical parameters were recorded by a blinded trained examiner (A.M.G.-D.L.F.) previous to surgery and 14 days after surgery. Twelve months after surgery, root coverage was registered in all patients.

-Surgical procedures

The surgical procedure consisted on the preparation of the recipient site by means of the bilaminar technique that was considered best in each case. Immediately after that, a graft was harvested from the palate, using the TD technique, as described by Edel (2), in 20 patients (control group) and the new technique we are describing, the “UPV/EHU technique”, in the remaining 20 patients (test group). The “UPV/EHU technique” starts with the elevation of a full thickness flap (FTF) in the palate, with an intrasulcular incision performed with a number 12 scalpel and preserving the papillae in the interproximal spaces. Then, the FTF will be dis-sected with a 15c scalpel, holding the flap with a tissues forceps and leaving the epithelium and a thin layer of connective tissue in the flap, so that the underlying connective tissue can be harvested (Fig. 1). In both groups, the graft was sutured into the recipient site with a resorbable suture (P.G.A. Rapid Arago®, Laboratorio Aragó SL, Barcelona, Spain) and then, both donor and recipient sites were sutured with a non-resorbable suture (Supramid® SMI-AG, St. Vith-Belgium).

Post-operative measures were identical for all the patients: betamethasone acetate/betamethasone sodium phosphate (Celestone Cronodose IM®, Merck Sharp & Dohme S.A., Spain) 6 mg in a single-dose intramuscular injection the day of the surgery, diclofenac sodium (Voltaren®, Novartis Farmacéutica S.A., Spain) 50 mg every 8 hours for 2 days and amoxicillin/clavulanic acid (Augmentine®, GlaxoSmithKline S.A., Spain) 875/125 mg every 8 hours for 7 days. Also, conventional oral hygiene techniques were interrupted in the surgical area for 15 days, and 0.12% chlorhexidine digluconate mouthwashes were prescribed twice a day for 6 weeks. Neither surgical dressings nor post-surgical stents were used in any case.

Sutures were removed 14 days after the surgery, and, at this visit, inflammation and infection were assessed by visual inspection, bleeding was recorded by asking the patient and postoperative pain in the donor site was measured with a visual analogue scale (VAS), in which 0 meant “no pain” and 5 was “the worst possible pain”. Pain was then recoded into three groups (“minimal pain” (1), “moderate pain” (2/3) and “severe pain” (4/5)), and the percentages for all the complications were calculated. The presence of necrosis in the palatal flap was clinically assessed with a periodontal probe, and recorded as the percentage of necrosis in the flap surface, classifying it into no necrosis, necrosis <30% of the total surface of the elevated flap or necrosis ≥30%.

-Statistical analysis of the results 

The statistical analysis was performed with the IBM SPSS v.20 software. Descriptive statistics were expressed as mean (SD) and percentages. Differences in the clinical trends (pain, bleeding, necrosis, infection) were analysed using a chi-square test (Fisher’s F exact if the sample was small). Results were considered as statistically significant when *p* <0.05.

## Results

40 patients took part in the study, 16 male and 24 female, with a mean age of 31.58 (SD: 8 years) [18-46 years]. A total of 57 Miller class I, II and III GR (20) recessions were treated, with 28 (70%) patients showing a single GR and 12 (30%) patients showing 29 multiple recessions being most of the latter class III GR (24/26) (92,31%). The descriptive analysis of both groups at baseline is summarized in  Table 1, showing a homogeneous distribution of all the variables for both groups. A mean root coverage of 89% was observed (98% for class I, 94% for class II and 80% for class III GR).

**Table 1:**
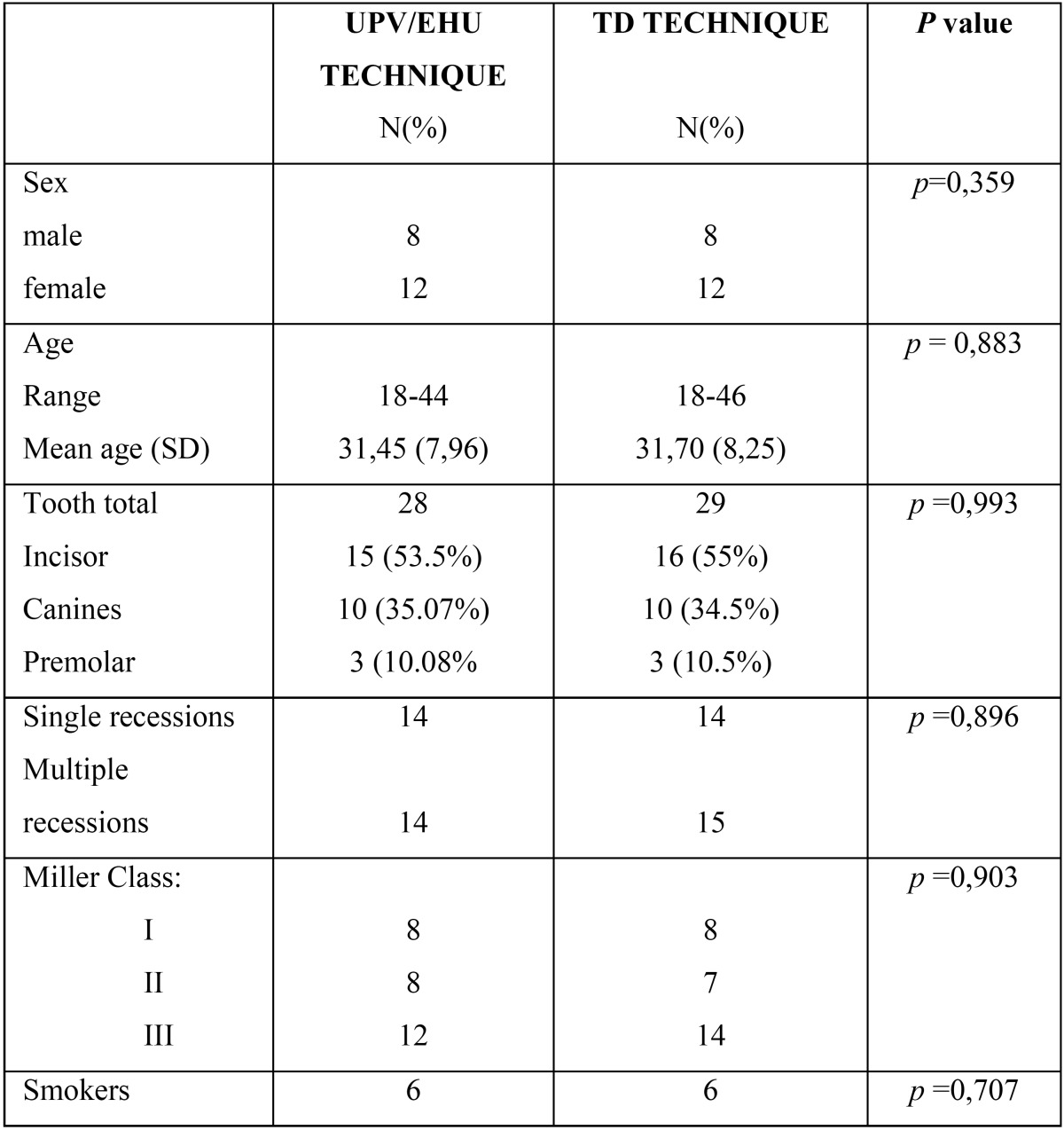
Descriptive analysis of both groups at baseline.

**Figure 2:**
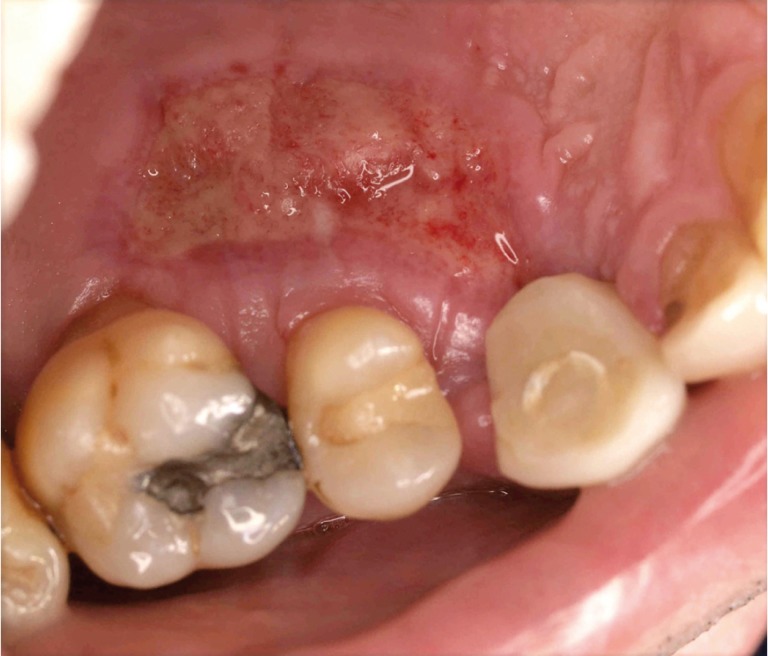
Necrosis in a patient of the control group.

**Figure 3:**
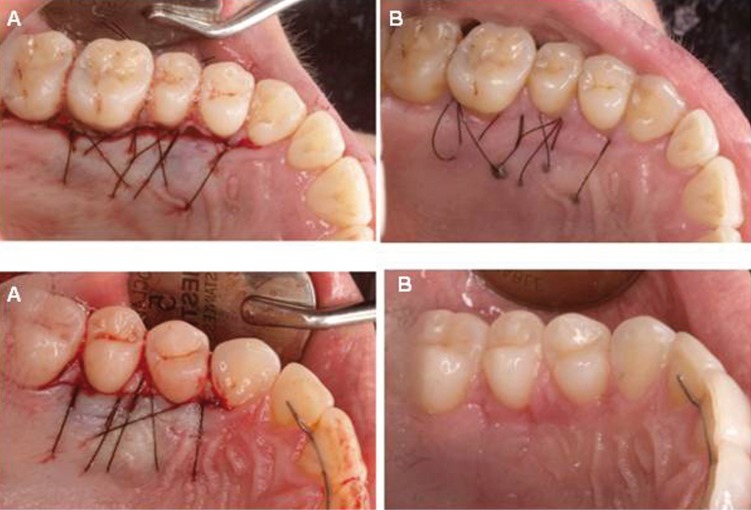
New technique (UPV/EHU technique). The day of the surgery (A) and 14 days after (B).

 Table 2 summarizes the number of patients who experienced pain (0-5), and the number of patients with bleeding, inflammation, infection and necrosis for both techniques (Figs.2,3). None of the patients in the test group experienced severe pain, with most of the patients referring none (35%) or minimal pain (30%), while, in the control group, 35% of the patients experienced severe pain, 5% of the patients referred a minimal pain, and only 10% referred no pain at all. There was a statistically significant difference favouring the test group in terms of absence of pain (*p*=0.001) and in terms of presence of pain (minimal, moderate, and severe pain) (*p*=0.001). With regard to the rest of complications, no infection or bleeding was observed in the test group, with 5% of the patients showing inflammation or necrosis ≥30%. In the control group, 15% of the patients presented with infection, and necrosis >30% was observed in 35% of the cases, which was significantly higher than in the test group (*p*=0.006). Besides, inflammation and bleeding were present in 25% and 15% of the patients, respectively.

**Table 2:**
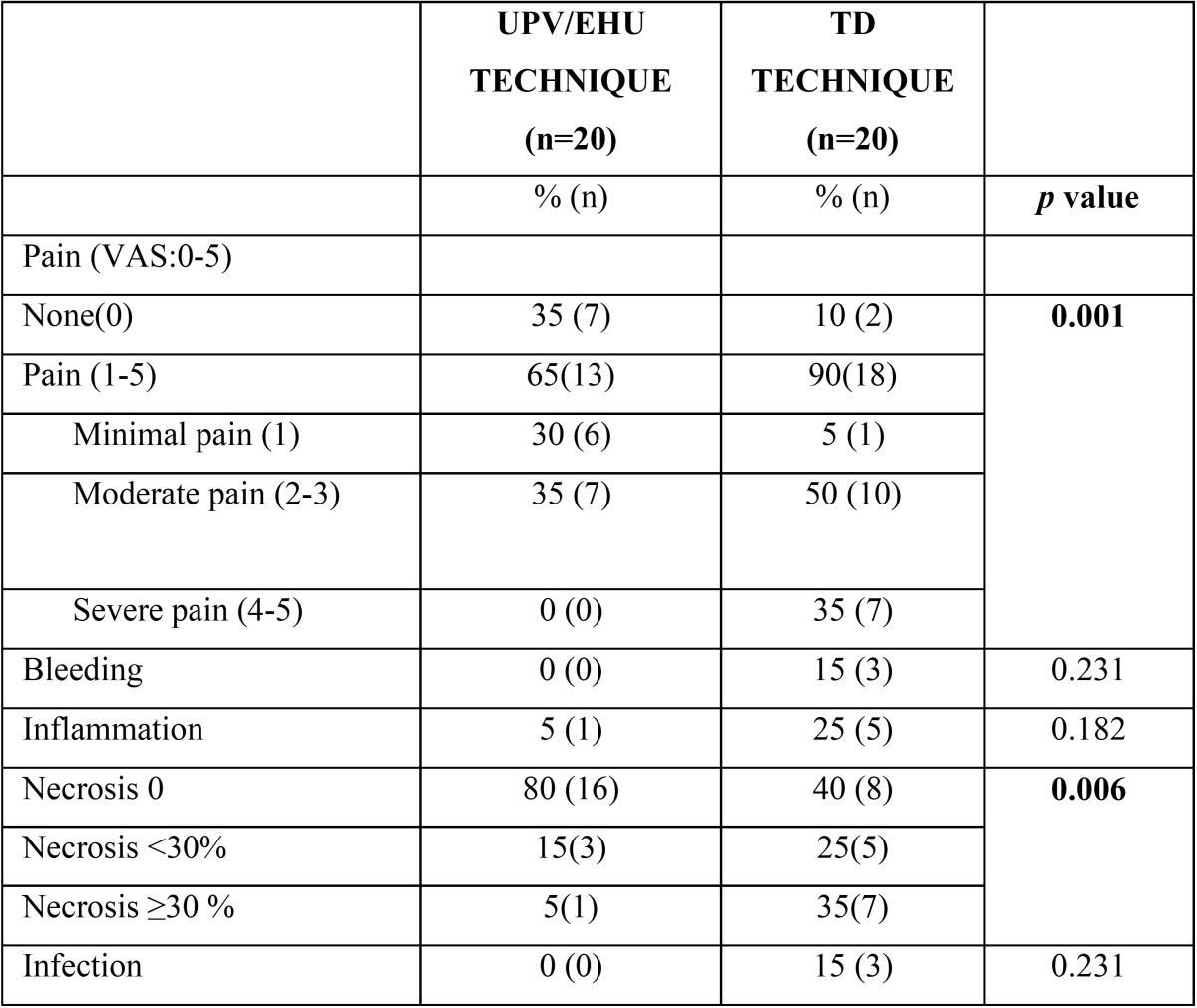
Postoperative complications in both groups.

## Discussion

Since Edel in 1974 (2) proposed the TD technique, different methods of harvesting a CTG have been described, in order to decrease the postoperative complications in the patients’ donor site (3-6,24). However, it should be kept in mind that any harvesting technique should grant the possibility of achieving an ideal graft, meeting the following characteristics (8): provide a proper size according to clinical requirements, achieve satisfying results in terms of alveolar ridge augmentation or coverage of gingival recessions, be an easy and fast-to-perform technique and cause as least as possible discomfort to the patient and as least as possible postoperative complications, creating a wound in the donor site that would heal quickly.

Only 3 studies (9,14,17) have compared the TD technique with other surgical choices (FGG, SI, modified-SI), while some other studies reported the results of an specific technique (10,12,15-16,18-19), or compared the FGG with PI or SI (8,11,13). Anyway, all of these studies showed that the most important complications after harvesting a graft would be pain, inflammation, bleeding, flap necrosis and infection in the donor site.

Concerning pain, in the present study, 35% of the patients in the TD group showed a severe pain, while in the test group no patient referred that degree of pain, which was statistically significant (*p*=0.001). Only 3 studies (9,14,17) have used TD as the negative control and their results are heterogeneous. Del Pizzo *et al.* (9) found that in the TD group 8% of the patients presented absence of pain at 14 days, but the authors did not inform about the degree of pain of the rest of patients; Zuchelli *et al.* (14) informed about intake of analgesics, showing that it was greater in the TD group, but it was not statistically significant, especially among patients experiencing primary intention healing without necrosis or infection. It should be noted that, in the present study, the patients in the control group who experienced a higher pain were also the patients who showed necrosis (28%), which was a relatively higher percentage than the 0% or 25% observed in previous publications (10,12, respectively). Although Fickl *et al.* (17) also showed that analgesics intake and duration of painkillers intake were lower in the test groups than in the TD group, when comparing pain levels they could not find significant differences.

Regarding inflammation, Harris (10), in his study on 500 patients treated with the PI technique, found inflammation in 5.4% of the cases, which is very similar to the percentage in our test group (5%), and clearly smaller than that reported by Griffin in 2006 (11), with a 34.9% of the patients treated with PI and a 18.6% of the patients treated with FGG showing signs of inflammation, or than the 30.3% observed by Roman *et al.* (15) in their patients treated with the SI technique.

Griffin *et al.* (11) found that the risk of having moderate to severe inflammation in the donor site was 3 times higher in smokers. Although Roman *et al.* (15) reported moderate inflammation in 30.3% of a sample that included smokers, the authors did not consider this issue. Also, Burkhardt *et al.* (18), although they only assessed pain, found that it was statistically significantly higher in thicker grafts and in smokers. On the other hand, Harris *et al.* (10), when studying the presence of complications, did not find any differences related to age, sex, smoking, location of the defect or the size of the graft. In the present study, there were smokers in both groups and no significant difference was found in any of the considered complications, in agreement with previous studies (10).

With respect to bleeding, Del Pizzo (9) found bleeding in 33% of the cases in which a FGG was performed, in 16% of the cases treated with the TD technique and in 8% of the cases treated with PI. On the other hand, Harris (10) found bleeding in only 2.2% of the 500 patients treated by means of the PI technique. Femminella *et al.* (19) reported no bleeding when comparing FGG and SI, which agrees with our results in the test group, where no stent or any other device was used in order to minimize such complication. Griffin *et al.* (11), when comparing FGG and PI techniques, found more bleeding in the first group (5.7% versus 1.2%), being those results better than the ones observed by Del Pizzo *et al.* (9) or by Harris *et al.* (10). In the present study, better results were too observed in the test group (0%) than in the control group (15%), similarly to Del Pizzo *et al.* (9).

Finally, concerning infection and necrosis, Harris (10) reported practically no infection, as it was observed in only 0.8% of the patients, and no signs of necrosis, when performing the PI technique. This is very similar to the results found with our proposed technique: no infection and necrosis >30% in 5% of the patients, which was statistically significant compared to the control group, where the presence of infection was 15% and necrosis > 30% was 35% ( Table 2). On the other hand, Yen *et al.* (12), in 2007, reported signs of infection and necrosis in 5% and 25% of the cases, respectively, after harvesting a graft with the SI technique. Zucchelli *et al.* (14), in their study on 50 patients treated with FGG or TD techniques, found necrosis in 28% of the patients in the TD group.

When harvesting connective tissue, it should be kept in mind that the different techniques do not only influence the postoperative complications, but also the quality and composition of the tissues.(25). Although the FGG technique is easier to perform than harvesting a CTG, it could have negative consequences, such as a greater postoperative pain and aesthetic alterations in the recipient site. Even when FGG are manually deepithelialized to achieve a connective tissue graft, and similarly to what happens when extraorally deepithelializing a subepithelial CTG, the remaining epithelial cell islets found by Harris in 2003 (26) could be the reason of the aesthetic complications (colour, texture, scar tissue…), so it should be studied in the long term (25). Also, Harris (26) reported that the quality of the tissues was irregular, obtaining a greater amount of lamina propria when more profound grafts were harvested. In the present study, satisfying results were observed in terms of mean root coverage, with nearly a 100% en Miller classes I and II and a slightly lower mean coverage in class III GR (80%). It should be noted that, in both groups, CTGs were harvested from profound areas.

It has been suggested that certain clinical parameters would be favourable indicators for achieving good results in the treatment of class III recessions (27). Esteibar *et al.* in 2011 (27) showed that the thickness of the graft was one of those factors.

Taking the aforementioned results into account, it seems that the newly described technique could meet the characteristics for obtaining the ideal graft, as proposed by Harris in 1997 (8), although it could be objected that it is a demanding technique in terms of the operator’s skills.

## Conclusions

Within the limits of the present study, it can be concluded that the use of the proposed technique minimizes the occurrence of postoperative complications in the donor site after harvesting a connective tissue graft from the palate, when compared with the trap-door technique. More studies are needed, which should be multicentric and with a bigger sample size, in order to confirm the results reported in the present study.

## References

[1] Sullivan HC, Atkins JH (1968). Free autogenous gingival grafts. 3. Utilization of grafts in the treatment of gingival recession. Periodontics.

[2] Edel A (1974). Clinical evaluation of free connective tissue grafts used to increase the width of keratinised gingiva. J Clin Periodontol.

[3] Harris RJ (1992). The Connective Tissue and Partial Thickness Double Pedicle Graft: A Predictable Method of Obtaining Root Coverage. J Periodontol.

[4] Hürzeler MB, Weng D (1999). A single-incision technique to harvest subepithelial connective tissue grafts from the palate. Int J of Periodontics Restorative Dent.

[5] Lorenzana ER, Allen EP (2000). The single-incision palatal harvest technique: a strategy for esthetics and patient comfort. Int J of Periodontics Restorative Dent.

[6] Kumar A, Sood V, Masamatti SS, Triveni MG, Mehta DS, Khatri M (2013). Modified single incision technique to harvest subepithelial connective tissue graft. J Indian Soc Periodontol.

[7] Chambrone L, Pannuti CM, Tu YK, Chambrone LA (2012). Evidence-based periodontal plastic surgery. II. An individual data meta-analysis for evaluating factors in achieving complete root coverage. J Periodontol.

[8] Harris RJ (1997). A comparison of two techniques for obtaining a connective tissue graft from the palate. Int J Periodontics Restorative Dent.

[9] Del Pizzo M, Modica F, Bethaz N, Priotto P, Romagnoli R (2002). The connective tissue graft: a comparative clinical evaluation of wound healing at the palatal donor site. J Clin Periodontol.

[10] Harris RJ, Miller R, Miller LH, Harris C (2005). Complications with surgical procedures utilizing connective tissue grafts: a follow-up of 500 consecutively treated cases. Int J Periodontics Restorative Dent.

[11] Griffin TJ, Cheung WS, Zavras AI, Damoulis PD (2006). Postoperative complications following gingival augmentation procedures. J Periodontol.

[12] Yen CA, Griffin TJ, Cheung WS, Chen J (2007). Effects of platelet concentrate on palatal wound healing after connective tissue graft harvesting. J Periodontol.

[13] Wessel JR, Tatakis DN (2008). Patient outcomes following subepithelial connective tissue graft and free gingival graft procedures. J Periodontol.

[14] Zucchelli G, Mele M, Stefanini M, Mazzotti C, Marzadori M, Montebugnoli L (2010). Patient morbidity and root coverage outcome after subepithelial connective tissue and de-epithelialized grafts: a comparative randomized-controlled clinical trial. J Clin Periodontol.

[15] Roman A, Balazsi R, Septimiu Cämpian R, Soancă A, Moldovan R, Sculean A (2012). Patient-centered outcomes after subepithelial connective tissue grafts and coronally advanced flaps. Quintessence Int.

[16] Zucchelli G, Mounssif I, Mazzotti C, Montebugnoli L, Sangiorgi M, Mele M (2014). Does the dimension of the graft influence patient morbidity and root coverage outcomes? A randomized controlled clinical trial. J Clin Periodontol.

[17] Fickl S, Fischer KR, Jockel-Schneider Y, Stappert CFJ, Schlangenhauf U, Kebschull M (2014). Early wound healing and patient morbidity after single-incision vs. trap-door graft harvesting from the palate—a clinical study, Clin Oral Invest.

[18] Burkhardt R, Hämmerle CH, Lang NP (2015). Self‐reported pain perception of patients after mucosal graft harvesting in the palatal area. J Clin Periodontol.

[19] Femminella B, Iaconi MC, Di Tullio M, Romano L, Sinjari B, D'Arcangelo C (2016). Clinical Comparison of Platelet-Rich Fibrin and a Gelatin Sponge in the Management of Palatal Wounds After Epithelialized Free Gingival Graft Harvest: A Randomized Clinical Trial. J Periodontol.

[20] Miller Jr PD (1985). A classification of marginal tissue recession. Int J Periodontics Restorative Dent.

[21] O'Leary TJ, Drake RB, Naylor JE (1972). The plaque control record. J Periodontol.

[22] Ainamo J, Bay I (1975). Problems and proposals for recording gingivitis and plaque. Int Dent J.

[23] Stillman PR (1932). A philosophy of treatment of periodontal disease. Dental Digest.

[24] Langer B, Langer L (1985). Subepithelial connective tissue graft technique for root coverage. J Periodontol.

[25] Zuhr O, Bäumer D, Hürzeler M (2014). The addition of soft tissue replacement grafts in plastic periodontal and implant surgery: critical elements in design and execution. J Clin Periodontol.

[26] Harris RJ (2003). Histologic evaluation of connective tissue grafts in humans. Int J Periodontics Restorative Dent.

[27] Esteibar JR, Zorzano LA, Cundin EE, Blanco JD, Medina JR (2011). Complete root coverage of Miller Class III recessions. Int J Periodontics Restorative Dent.

